# Early Diagnosis of Abusive Head Trauma to Avoid Repetitive Shaking Events

**DOI:** 10.1177/2333794X211067037

**Published:** 2022-02-20

**Authors:** Sarah Depallens, Celine Favrod, Philippe Maeder, Diego San Millan, Jean-Jacques Cheseaux

**Affiliations:** 1 Lausanne University Hospital, Lausanne, Switzerland; 2 Valais Hospital, Sion, Valais, Switzerland

## Introduction

Since 2009, Abusive Head Trauma (AHT) is preferred to the term of Shaking Baby Syndrome (SBS) to characterize the type of injury rather than the mechanism.^1^ It is a severe form of abuse in infants and young children. AHT is the most common cause of child abuse mortality in children less than 2 years old. It was originally described in children younger than 2 years old, and is more frequently detected between 3 and 8 months.^1^ Approximately 65% of the survivors will present neurologic sequelae, such as motor or cognitive impairment or mental retardation.^1^ In a Swiss follow-up study (2002-2007), the incidence was 14/100 000 (2002-2007)^2^ but is estimated to be 35/100 000 in the first year of life.^1^

Neurologic symptoms, subdural hemorrhage (SDH) and retinal hemorrhage (RH) are 3 characteristic findings of AHT.^1^ The diagnosis is based on a combination of imaging and physical findings.^1,2^

SDH is the most commonly reported abnormality in the case of AHT.^1^ RH is common in cases of AHT and has a good predictable value. Characteristics differ in case of abusive versus accidental trauma.^3^ In case of AHT, RH are often bilateral, pre- intra- and subretinal and involve the entire retinal surface.^3^

Only a few studies have focused on missed cases of AHT.^4,5^ An important issue is the under-recognition and under-reporting of AHT.^6^ In severe forms, the neurological symptoms, such as apneas, convulsions, or coma will easily lead the pediatrician toward brain injury investigations. However, in less severe forms, a transient drop in alertness may appear, or non-specific symptoms (pallor, vomiting, irritability, eating, or sleeping disorders) may mislead physicians to consider other common diagnosis of a common pediatric illnesses. Multiple consultations often occur before the diagnosis of AHT^1^ is established. In a study investigating the medical files of children who died from AHT, almost 20% of them had seen a medical professional within the month before.^7^ It is thought that about one third of AHT is initially misdiagnosed.^1^

Based on caregiver confessions, shakings are repetitive in 55% of the time.^8^ In medical practice, in most cases, there are no confessions nor good explanations for the trauma. Signs of repetitive shakings are controversial; therefore, repetitive shakings are difficult to prove. Nevertheless, repetitive shaking can be evaluated from the type and locations of subdural hematoma on MRI, with mixed intensity hematoma in different locations being highly suggestive of repetitive AHT.^9^

There are still fewer studies on missed diagnoses of infants with AHT. Targeted studies of diagnostic approaches to these situations would improve early detection of abuse and lead to a better understanding of the clinical course of non-recognized cases of AHT. The aim of the study was 2-fold. First, to analyze the medical cases of infants admitted to the Lausanne University Hospital for Abusive Head Trauma (AHT) between 2002 and 2015 and to determine the proportion of multiple shaking events. Second, to determine whether these patients had been seen previously by a pediatrician for non-specific neurological symptoms, and whether AHT could have been misdiagnosed at that time.

## Method

### Setting

The data were collected from patients’ data base of University Hospital. All cases containing one of the following keywords “Shaking Baby Syndrome,” “Abusive Head Trauma,” “Subdural hemorrhages,” or “Retinal Hemorrhages” were eligible for the actual study. The first author (SD) selected cases when inclusion criteria were present and exclusion criteria absent.

This research was approved by the Ethics Committee of the University School of Medicine.

### Inclusion Criteria

Patients who were 2 years old or younger diagnosed with certain or likely cases of AHT between 2002 and 2015 at the *University Hospital* were included in this retrospective, observational study. We used the criteria of AHT defined by the Hautes Autorités de Santé (HAS)^10^: “The diagnosis of non-accidental shaking head injury is certain in cases of: Multi-focal SDH with clots at convexity (vertex) reflecting the rupture of bridging veins, or multi-focal SDH and HR of any kind, or unifocal HSD with cervical and/or spinal cord injuries. The diagnosis of non-accidental shaking head injury is likely in cases of : multifocal HSD even without any other lesions, or unifocal SDH with intraretinal HR limited to the posterior pole, or HR touching the periphery and/or several layers of the retina, whether single or multiple layers of the retina.”

Patients with available CT and/or MRI were included.

### Exclusion Criteria

Cases with a metabolic disease, a coagulation disorder or an accidental trauma that could explain the SDH were excluded.

### Collected Data

Age, gender, previous emergency consultations before admission, symptoms, and initial diagnosis at the time of the first consultation, history and clinical examination at hospital admission, and AHT investigations (neuroimaging, fundus examination, skeletal survey) were collected in the medical file of the patients.

### Imagery Analysis to Diagnose Repetitive Shaking Events

Two independent board-certified senior neuroradiologists from 2 different hospitals reviewed Cerebral Computerized Tomography (CT) and Magnetic Resonance Imaging (MRI), anonymously and without indications on clinical history.

CT and MRI density and signal characteristics of SDH were used to establish the age of the SDH. Density/signal abnormalities consistent with chronological different SDH were considered confirmatory for repeated shaking episodes.

Since criteria to date of subdural hematoma are controversial in the literature, we added the fact that simultaneously acute and chronic hematomas must be present in a different location to make the assessment of highly probable repeated shaking events.^9^ Repeated shaking events were considered only if the diagnosis of multiple SDH of different ages was concordant between both neuroradiologists.

## Results

This study included 19 patients, 10 girls (53%) and 9 boys (47%). None of the patients presented a coagulation disorder or a metabolic diseases that could explain neurologic symptoms and/or SDH. The mean age of patients was 4.8 months (2-9 months). All results are described in Table 1.

**Table 1.** Details of Cases Included in the Study.

Gender	Age (months)	Delay prevention consultation (day)	Symptoms of previous consultation	Diagnosis of previous consultation	Symptoms at admission	Radiologists’ ratings of subdural hemorrhages (SDH)1. R12. R2Radiology	Retinal hemorrhages (RH)	Other findings
F	4	75	VO, IRR	IU	VO, IRR	1.2. Different ages	UNI	Head bruise
						CT	ML	
M	3	None	—	—	HYPO, LOC	1. Different ages	UNI	—
						2. Not sure		
						MRI		
M	5	10	HYPO, LOC	GE	HYPO, LOC	1. Different ages	BI	—
						2. 1 age	ML	
						MRI		
M	4	None	—	—	HYPER, LOC	1. Different ages	BI	—
						2. 1 age	ML	
						CT and MRI		
F	4	28	CONV, LOC	Intuss.	LOC, DEATH	1.2. Different ages	BI	—
						CT	ML	
M	8	15	VO, AOC	IVRS	VO, IRR	1.2. Different ages	BI	—
						CT and MRI	ML	
F	9	None	—	—	VO, IRR	1.2. Different ages	BI	—
						CT and MRI	ML	
M	8	None	—	—	IRR, LOC	1.2. 1 age	UNI	—
						CT and MRI		
F	5	14	VO, AOC	GE	VO, IRR	1.2. Different ages	BI	—
						MRI	ML	
F	7	Many	CRY	—	LOC, DEATH	1.2. One age	BI	—
						CT	ML	
M	2	None	—	—	LOC	1. Different ages	BI	Old rib fracture
						2. Not sure	ML	
						MRI		
F	5	15	CONV, VO	GE	CONV	1.2. Different ages	BI	—
						CT and MRI	ML	
M	3	No data	—	—	HYPO, AOC	1.2. Different ages	BI	—
						CT and MRI	ML	
F	6	11	VO, IRR	GE	VO, IRR	1.2. Different ages	No	Rib fractures, clavicule fracture
						CT and MRI		
M	4	None		—	AOC	1.2. Different ages	BI	—
						CT and MRI	ML	
F	2	5	CRY	—	HYPO, CONV	1.2. Different ages	BI	Rib fractures, tibia fracture head, thorax, and legs bruises
						CT and MRI	ML	
F	4	None	—	—	CONV, VO	1.2. 1 age	BI	Face bruises
						CT and MRI	ML	
F	3	4	VO, CRY		CONV, IRR	1. Different ages	BI	Old cranial fractures
						2. Not sure	ML	Face bruises
						MRI		
M	5	3	VO, IRR	GE	VO, IRR	1.2. Different ages	BI	Rib fracture
						MRI	ML	

Abbreviations: R1, first independent neuroradiologist expertise on SDH; R2, second independent neuroradiologist expertise on SDH; M, male; F, female; VO, vomiting; IRR, irritability; HYPO, hypotonia; LOC, loss of consciousness; AOC, alteration of consciousness; CRY, crying; CONV, convulsions; UI, urinary infection; GE, gastroenteritis; Intuss., intussusception; IVRS, infectious viral respiratory superior; SDH, subdural hemorrhages; R1 or 1, neuroradiologist 1; R2 or 2, neuroradiologist 2; RH, retinal hemorrhages; BI, bilateral; UNI, unilateral; ML, multiple layers; no, no ophthalmology examination.

Hospital admission was motivated in a few cases by recurrence of irritability and vomiting or by worsening status, with altered state of consciousness, hypotonia, or seizures, with all patients presenting neurological symptoms at admission. These clinical findings lead to neuroimaging, fundoscopic examination, and skeletal survey, which made the diagnosis of AHT. Altered consciousness was the most frequent symptom, present in 9 patients, with complete loss of consciousness in 7. Two patients died soon after admission. Other clinical symptoms included irritability (8/19), vomiting (7/19), convulsions (4/19), hypotonia (4/19), and hypertonia (1/19). Many patients presented several of these symptoms.

Emergency CT was performed in all patients. In 2 patients, demise occurred soon after admission before a head MRI could be obtained. The 17 remaining patients obtained an MRI study. All of them presented subdural hemorrhages (SDH). About 3 of 19 presented only one SDH, so different SDH ages could not be determined. In 16 of 19 patients (84%), HSD were identified in separate locations. In 5 of these (5/16; 31%), the neuroradiologists were not able to agree on repetitive or single events based on the signal characteristics of the SDH. Eleven of the 16 patients (69%) with HSD in separate locations presented different signal characteristics consistent with chronological different HSD and repeated shaking events. If all 19 patients are considered, imaging was consistent with repeated shaking events in 58% of the cases.

All patients had a fundoscopic examination. Retinal hemorrhages were present in 18/19 patients (95%). They were bilateral and extended to multiple layers of the retina in 15/18 cases (83%), and unilateral in 3/18 cases. In addition, 7/19 infants had associated lesions, such as fractures or bruises at the time of diagnosis of AHT. Five patients (26%) had external bruises, 4 of which were located on the head. All patients had partial to extended skeletal survey and/or bone scintigraphy. Skeletal survey revealed 7 fractures (4 ribs, 1 clavicle, 1 tibia, and 1 skull) in 5/19 patients (26%), with 2 presenting a combination of different fractures.

Eight of the 11 patients (73%) with confirmed repeated shaking events on imagery had been seen in emergency consultation by a pediatrician on average 21 days before the diagnosis of AHT (min 3 days; max 75 days). Symptoms were varied (vomiting, irritability, hypotonia, loss of consciousness, alteration of consciousness, crying, convulsions) and consistent with non-specific neurological symptoms. Diagnosis of this previous consultation included suspected gastroenteritis, viral respiratory superior infections, urinary infection, and intussusception.

## Discussion

This observational, retrospective study investigated infants who were treated at University Hospital with a diagnosis of AHT. Based on CT and MRI and the evaluation of SDH, the main finding of the study was that 58% of the cases were identified as victim of repeated shaking event. If only the cases with SDH in different locations are considered (16/19 patients: 82%), repeated shaking events were identified on 68% (11/16) of the cases.

Our population aged from 2 to 9 months, in keeping with previous clinical observations reporting that two-third of AHT patients are under 6 months old.^11^

The medical files of the 11 patients with high suspicion of repeated shaking events revealed that 8 (73%) of them had an emergency consultation preceding the final diagnostic of AHT. Radiologic diagnosis of multiple events of AHT is therefore supported by non-specific symptoms observed during previous medical consultations. In the case of AHT, bruises or fractures are frequent associated findings. Given that infants haven’t acquired the capacity to walk, any bruises discovered within the first months of life should alert medical staff to AHT. AHT carries a risk of severe sequelae including epilepsy, developmental delay, blindness, cerebral palsy and death (10% in this study). Early detection of AHT is therefore essential to prevent severe sequelae and death.

This study shows that the diagnosis of AHT can be difficult, as infants can present non-specific symptoms that could engender a wrong diagnosis and non-recognition of AHT. Informing and training medical and nursing staff is thus essential in order to decrease the risk of misdiagnosis. Pediatricians should include in the list of differential diagnoses the possibility of AHT when an infant is presenting non-specific symptoms.

This study has several limitations. First, the small number of cases does not allow for statistical analysis. Second, due to existing controversies regarding the identification of lesions of different ages on brain imaging, we adapted the methodology and we retained as repeated shaking events only the cases where the 2 neuroradiologists were unanimous in their diagnosis. This could cause an underestimation of multiple shaking events cases. Imaging data was also inhomogeneous due to the retrospective nature of the study.

Future research should focus on identifying clinical symptoms associated with child maltreatment situations in order to improve early detection of AHT.

## Conclusion

This observational, retrospective study found that AHT should be systematically part of the differential diagnosis when an infant presents non-specific neurological symptoms (vomiting, excessive crying, increase of the head circumference, developmental delay).^12^ Information and training of medical staff is therefore essential in order to be able to better detect AHT and thus avoid a new shaking episode that can have serious consequences on the health and life of the patient.
